# PacBio sequencing output increased through uniform and directional fivefold concatenation

**DOI:** 10.1038/s41598-021-96829-z

**Published:** 2021-09-10

**Authors:** Nisha Kanwar, Celia Blanco, Irene A. Chen, Burckhard Seelig

**Affiliations:** 1 Department of Biochemistry, Molecular Biology and Biophysics, University of Minnesota, Minneapolis, MN 55455 USA; 2 BioTechnology Institute, University of Minnesota, St. Paul, MN 55108 USA; 3 Department of Chemical and Biomolecular Engineering, University of California, Los Angeles, CA 90095 USA

**Keywords:** Biochemistry, Biotechnology, Sequencing, Experimental evolution, Molecular evolution, Bioinformatics, DNA sequencing, Next-generation sequencing, RNA sequencing

## Abstract

Advances in sequencing technology have allowed researchers to sequence DNA with greater ease and at decreasing costs. Main developments have focused on either sequencing many short sequences or fewer large sequences. Methods for sequencing mid-sized sequences of 600–5,000 bp are currently less efficient. For example, the PacBio Sequel I system yields ~ 100,000–300,000 reads with an accuracy per base pair of 90–99%. We sought to sequence several DNA populations of ~ 870 bp in length with a sequencing accuracy of 99% and to the greatest depth possible. We optimised a simple, robust method to concatenate genes of ~ 870 bp five times and then sequenced the resulting DNA of ~ 5,000 bp by PacBioSMRT long-read sequencing. Our method improved upon previously published concatenation attempts, leading to a greater sequencing depth, high-quality reads and limited sample preparation at little expense. We applied this efficient concatenation protocol to sequence nine DNA populations from a protein engineering study. The improved method is accompanied by a simple and user-friendly analysis pipeline, DeCatCounter, to sequence medium-length sequences efficiently at one-fifth of the cost.

## Introduction

Over the past decade, advances in DNA sequencing technology have accelerated at an unprecedented rate^1^. Next generation sequencing (NGS) approaches have improved our ability to study genomes and large libraries of DNA for a fraction of previous costs^2–4^. Consequently, advances in DNA sequencing technology bring benefits to many fields of research, including molecular engineering^5^. In molecular engineering, large mutational libraries of DNA encoding protein variants are frequently used to select, screen, or probe for specific properties or activities^6,7^. Such studies face challenges resulting from the limitations of DNA-sequencing technologies, in particular when reliant on laborious and expensive Sanger sequencing-based methods^8,9^. However, NGS technologies have enabled simultaneous high-throughput sequencing of millions to billions of DNA molecules and allowed for a more comprehensive analysis of selection and screening outputs, thereby probing a greater proportion of the potential sequence space^10,11^. Notable achievements include the ability to investigate local fitness landscapes of proteins or nucleic acids, to perform in-depth structure to function studies, and to analyse local and global regions of proteins^12–15^.

At the forefront of NGS technologies are Illumina sequencing methods, which can sequence 150–600 base pairs (bp) and up to one billion sequences^16^. Furthermore, emerging third generation sequencing technologies, such as PacBio and Oxford Nanopore, can sequence much longer reads^17,18^. Such long-read sequencing techniques are starting to gain traction because they remove the gene assembly process, or polishing, necessary when handling short reads^3^. This is particularly important when the DNA sample contains similar sequences, as the assembly of short reads can lead to chimeric contigs. Long reads are also less susceptible to issues caused by GC rich, complex, repetitive, or heterozygous regions as well as structural variations in the DNA^19–22^.

Long-read sequencers are currently unable to compete with the sequencing depth (up to billions of sequences) or sequence accuracy (0.1–1% error per base) possible with Illumina technology^23^. On the other hand, Illumina sequencing is limited by its ability to only sequence relatively short reads (< 600 bp)^1–3^. This shortcoming has often constrained protein engineering studies to focus on only small length nucleic acids/protein libraries, or to investigate mutations only in small regions of larger protein or nucleic acid sequences^24^. The study of larger regions of nucleic acids or translated genes (> 600 bp) remains challenging with current sequencing technology. To overcome these challenges, smaller regions of interest are often sequenced in-depth using Illumina and supplemented with some full-length sequencing by complementary long reads via the Sanger or PacBio method^25^. In this context, it has become simple to assess the consequences of individual mutations, but epistatic relationships between sets of discrete mutations remain challenging to discern^26,27^. Other methods have been developed to sequence DNA molecules of > 600 bp, using Illumina high-throughput sequencing. Generally, these methods require a unique molecular identifier (UMI) attached to each DNA molecule. In a study by Turchaninova et al.^28^, DNA molecules of 750 bp were sequenced using asymmetric paired-end sequencing. Each molecule was appended with a UMI tag, amplified, and then sequenced from both ends with the 400 + 100 nt paired-end sequencing method. The resulting ~ 50 bp overlapping region and the UMI were used to identify, error-correct, and assemble in silico the two halves of the DNA molecule to obtain the full-length 750 bp sequence. In a paper by Sarkisyan et al.^29^, DNA libraries and an intrinsic C-terminal UMI were built into a plasmid vector. The C- and N-terminal halves of each DNA molecule were sequenced separately by carrying out site-specific digestions to either isolate the C-terminal region with the intact UMI, or to remove the C-terminal region so that the N-terminal region could self-ligate to the C-terminal UMI. The UMI sequence was used to identify and assemble the two halves of each DNA molecule, producing the full-length sequence. Both examples demonstrated a novel approach to sequence longer (> 600 bp) DNA molecules. However, both methods are limited to sequencing fragments no longer than 800 bp due to the length limitations of Illumina paired-end sequencing. Furthermore, the methods rely on developing sophisticated sample preparation, the efficiency of the UMI tags, and additional bioinformatic steps. Finally, both methods require a series of amplification and modification steps, which can change the original sample population and result in sequence-specific biases^19,30^.

We sought to demonstrate a simple, robust method to sequence large populations of protein mutants with gene lengths of larger than 800 bp. In principle, long-read sequencing is already possible with third generation PacBio and Oxford Nanopore technologies. However, these third-generation sequencers suffer from poor sequencing accuracy of only 90% (equal to a quality score of Q10), compared to Illumina sequencing accuracy of 99.9% (Q30)^23,31^. In protein engineering studies, sequencing accuracy is critical, as single base pair point mutations can dramatically change the properties of the translated protein^32^.

PacBio technology can achieve a sequencing error rate comparable to Illumina sequencing through a method called circular consensus sequencing (CCS)^33,34^. The CCS method by PacBio creates a “SMRTbell” template by attaching ssDNA hairpin adapters to the target dsDNA, allowing the polymerase to sequence each strand of the dsDNA target multiple times. The process results in one continuous long read (CLR) consisting of multiple subreads (passes) of the target sequence^33^. Unlike Illumina sequencing, the PacBio sequencing error is unbiased and so the overall accuracy of the sequence can be increased through multiple reads of the same molecule^35^. The sequencing accuracy is concurrently determined by the length of the DNA and the number of passes possible by the polymerase. CCS allows accurate long-read sequencing of up to 60,000 bp^36,37^. However, the major caveat for PacBio Sequel I instrument is that the throughput or sequencing depth is limited to ~ 100,000–300,000 sequences^38^. Schlecht et al., previously increased the sequencing output of PacBio using a method called ConcatSeq^39^. This method concatenated several DNA fragments using Gibson assembly to produce one longer DNA amplicon for sequencing. This longer sequence was then attached to PacBio SMRTbell adapters and sequenced. The study used ConcatSeq to sequence DNA fragments of 80–800 bp in length by concatenating them into larger amplicons, increasing the sequencing output of PacBio by utilizing its long-read sequencing potential. The ConcatSeq method resulted in amplicons with mixed directionality and a range of fragment sizes due to the inability to control the degree of concatenation, causing an undesired reduction of the sequencing output. Nevertheless, ConcatSeq demonstrated a proof of concept, and—if optimised—could provide a real benefit for sequencing DNA fragments with > 600 bp and high accuracy^39^.

A protein engineering project in our group served as motivation to find a method to sequence several large populations of ~ 870 bp genes with high accuracy, high depth, but at a low price. These populations encoded protein libraries stemming from stages of a directed evolution campaign^40,41^. It was important to sequence each population to sufficient depth in order to discern enrichment of specific sequences and enable the analysis of relationships between specific positional mutations. Inspired by ConcatSeq^39^, we developed an improved concatenation method. We redesigned the concatenation procedure to control the rate and directionality resulting in a high-quality sample for PacBio sequencing. We also optimised the sample preparation process to enable reliable and efficient concatenation. In addition, we developed a simplified sequencing data analysis pipeline. We were able to sequence nine different sample populations and discern sequence-specific changes across these populations using a single PacBio flowcell. In doing so, we were able to successfully increase the output of a PacBio CCS run by up to fivefold, or reduce the price to one-fifth the cost compared to the standard non-concatenated protocol. This simple method can be directly applied to any library, providing easy, routine access to sequencing > 600 bp medium-length DNA molecules efficiently at a greatly reduced cost without compromising on the quality of sequencing data.

## Results

### Strategy and design of the method

**Figure 1:**
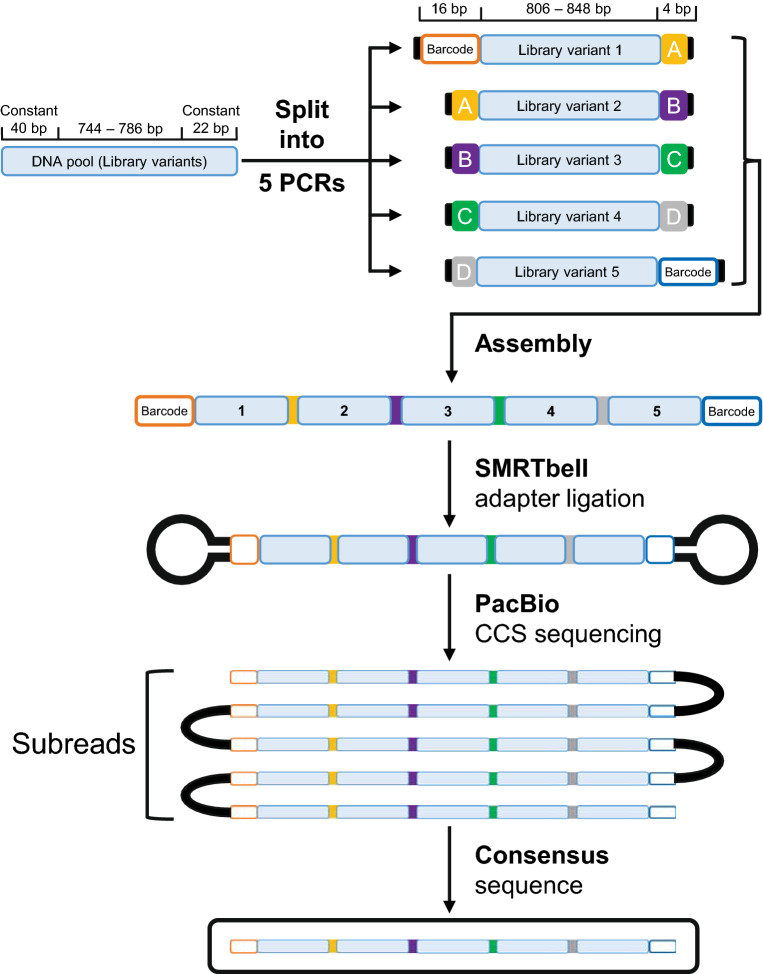
Overview of the concatenation procedure. The DNA pool to be sequenced was split into five PCR reactions with distinct primer sets, producing library variants 1–5. The PCR reactions also appended the variants with complementary BsaI restriction sites, which contained the four unique scar sequences (**A**–**D**) (yellow, purple, green, grey). The complementary overhangs are shown as black rectangles. Sample-specific barcodes were attached to the 5′-end of library variant 1 and the 3′-end of variant 5. The design enabled controlled and directional assembly of the five library variants upon Golden Gate assembly. The different barcoded concatemers were all combined and SMRTbell hairpin adapters were ligated to the barcodes of each amplicon. PacBio CCS sequencing and the SMRTbell hairpin adapters allowed continuous reads of the large concatemer amplicons producing several subreads. The consensus of the subreads produced high fidelity sequence reads.

We sought to develop a simple method to increase the sequencing capability of PacBio CCS to sequence several diverse DNA libraries ~ 870 bp in length that encoded protein variants originating from a directed evolution campaign. To achieve an increase in the throughput of a PacBio sequencing run, we assembled individual genes by concatenation, producing larger DNA amplicons (Fig. 1). We limited the assembly of amplicons to contain five genes each to ensure that the polymerase will make at least three passes of each of the resulting ~ 5,000 bp amplicons during the CCS sequencing run. The consensus of these three subreads corrects random sequencing errors, thereby providing a sequencing accuracy of at least 99%. If a gene of a different length was to be concatenated, the number of genes per amplicon would have to be adjusted to yield a similar final amplicon length. The concatenation of the genes was carried out using Golden Gate assembly, which utilises Type IIS restriction enzymes^42,43^. Type IIS enzymes cleave DNA distal from its recognition site, subsequently removing the recognition site from the sequence^44^.

Before assembling the five genes, the DNA libraries were first purified by agarose gel electrophoresis to remove any species of incorrect length. We then divided each purified DNA library into five aliquots. We amplified each aliquot by PCR using five different pairs of primers yielding the library variants 1–5 (Supplementary Table S1). The primers were designed to overlap with the terminal constant regions of the encoded protein variants resulting in a slightly reduced size of the sequenced protein variants of 806–848 bp. The primers also introduced flanking BsaI restriction sites with the appropriate overhangs. A 16 bp sample-specific barcode was added during PCR to the 5′ end of library variant 1, and the 3′ end of library variant 5, providing a unique identifier to differentiate sample populations (see Supplementary Table S2 for barcode specific primers). All PCR primers were designed to have similar melting temperatures and GC-content to prevent primer dimers and mispriming events. The resulting PCR products (library variants 1 to 5) represented the genes one to five of the final assembled concatemer amplicon (Fig. 1).

We designed the primers such that four unique 4 bp “scar” sequences A to D (see Supplementary Table S1) were located at the connection between each of the five library variants after the Golden Gate assembly. Each scar was chosen based on previous studies that demonstrated increased assembly efficiency for particular sequences^45^. These scars enabled us to easily identify the individual genes (library variants 1 to 5) in the full-length amplicon.

To assemble the full-length amplicon, we mixed equimolar amounts of the amplified library variants 1 to 5. The Golden Gate reaction mix contained the BsaI restriction enzyme to produce overlapping flanking regions and T4 DNA ligase to ligate each of the variants in a one-pot reaction, producing the final concatenated amplicon ‘12345’. Facilitated by the unique pair of barcodes that flank each final amplicon, several DNA libraries were pooled and sequenced together on a single PacBio run.

Our initial attempts, following standard protocols to assemble the full-length 5 × amplicon, yielded mostly partially assembled products, which made it impractical to obtain the 1 µg of the final amplicon as required for PacBio sequencing (Supplementary Fig. S1). Most published protocols for Golden Gate assembly are designed for the assembly of fragments from circular vectors, but not from linear dsDNA like in our case^46^. We therefore optimised each stage of the concatenation process to ensure sufficient yield of the fully assembled 5 × amplicon.

### Optimization of concatenation

To reduce the impact of potential PCR bias in sequencing, it was important to optimise the amplification of the library variants 1 to 5 to avoid mispriming events, non-target amplification, overamplification, and to minimise any impact of the original PCR template upon assembly. We first identified an annealing temperature that produced a similar amount of the target amplified product for each of the primers sets without amplifying non-target PCR species. This was important to minimise any potential bias between library variants 1–5. A temperature gradient established that an annealing temperature of 54 °C was optimal for all primer sets. Amounts of > 10% of the original template DNA in the final amplified DNA were found to be detrimental to assembly efficiency. The template appeared to function as a non-competitive inhibitor towards the restriction and/or ligase enzymes, leading to partial or improper assembly of the full-length amplicon and producing a smear rather than discrete bands for the assembled products (Supplementary Fig. S1). The PCR amplification was therefore optimised to produce sufficient product with the least number of PCR cycles to reduce potential bias, while keeping the amount of original template below 10%. The PCR product was purified by AMPure XP beads to remove any residual primers. About 2 µg of each of the purified library variants (1 to 5) was needed for the subsequent procedures.

Assembly of the library variants to produce the 5 × concatenated amplicon was carried out in a one-pot reaction with the BsaI enzyme, T4 DNA ligase and an equimolar mix of all five library variants 1 to 5. The enzyme BsaI was chosen because it produces a small overhang of only 4 bp that is still sufficient for efficient ligation^47–49^. However, alternative type IIS restrictions enzymes could be used, especially in case the gene of interest already contained a BsaI recognition site. Previous research has shown varying assembly yields using different kits or different enzyme vendors. Therefore, we compared several commercially available enzymes including T4 DNA ligase, T7 DNA ligase, BsaI, BsaI v2, and Eco31I. For our assembly, we found the NEB Golden Gate assembly kit to be most efficient, whereas enzymes from Promega and Thermofisher showed slightly lower efficiency (see Supplementary Fig. S2). However, all enzyme combinations showed partial assembly. The yield of the 5 × amplicon was further improved by optimizing protocol details. The reaction is often cycled between 37 °C (optimal temperature for digestion enzyme) and 16 °C (optimal for ligase reaction) for a total of 30 cycles. When the time at each temperature was increased from 1 to 5 min and the number of cycles was increased to 50, the production of the fully assembled amplicons increased. (Supplementary Fig. S2).

**Figure 2:**
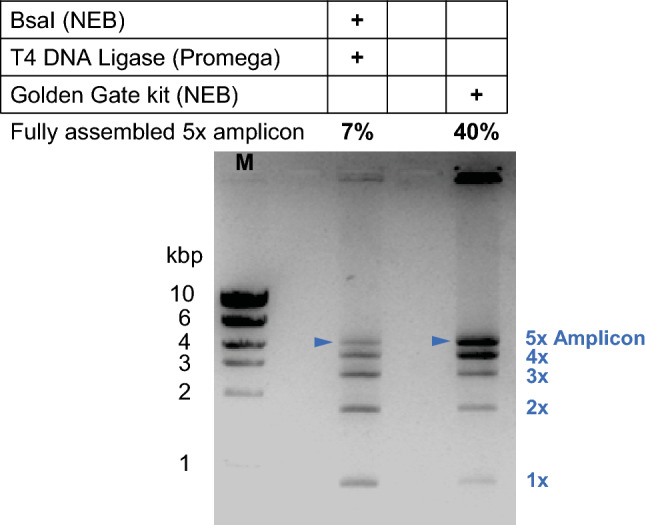
An example agarose gel showing the efficiency of concatenation for library variants 1–5 using enzymes from different commercial sources. Performance was quantified for each reaction by the percentage of fully assembled 5 × amplicon (marked by blue triangles). The NEB Golden Gate assembly kit was shown to concatenate the library variants more efficiently, increasing the amount of fully assembled product to 40%. A more detailed comparison can be seen in the Supplementary Fig. S2.

The combined optimization of the PCR and assembly reaction conditions improved the yield of the fully assembled 5 × amplicon from initially 7% to 40% but did not completely remove the partially assembled products of 1 × to 4x (Fig. 2). This is not surprising since the Golden Gate protocol usually assembles genes from vectors, which has been shown to have higher efficiency than the assembly of linear dsDNA. Furthermore, the efficiency in many of those studies was assessed indirectly through transformation efficiency^43,50^.

To enable efficient sequencing, the fully assembled 5 × amplicon was purified from the shorter concatemers using size separation by agarose gel electrophoresis. The DNA recovery from gel extraction is usually below 50% and the recovered DNA is often dilute and contaminated with residual EDTA or guanidine salts. However, the efficiency of PacBio sequencing is highly sensitive to the purity and quality of the DNA sample. Therefore, we included additional washing steps to reduce potential contaminants. The DNA samples were further purified and concentrated using AMPure XP beads (Beckman Coulter). The assembly reaction was scaled up to attain enough fully assembled 5 × amplicon for each gel extracted DNA library. We quantified the sequencing samples with PicoGreen and performed a size analysis via Agilent Bioanalyzer.

We applied this strategy to nine separate DNA library samples S1-S9, which were the output of different directed evolution experiments performed with the same starting library. The goal was to compare the protein sequence populations across the different experiments to understand the dependence of specific mutations on changing experimental conditions. After purification and quantification, the nine concatenated DNA samples were combined to a single pool at a ratio that reflected the relative sequencing depth we desired for each sample (Table 1). Finally, we submitted the combined, yet individually barcoded, sequencing samples for PacBio sequencing (Supplementary Fig. S3).

**Table 1:** Recovery of sequences during the post-sequencing data processing. All nine different DNA library samples, each containing a unique sample specific pair of barcodes, were combined and sequenced in a single flowcell.

Sample	Sample barcodes		Fraction of sample	Demultiplexed		Deconcatenated		Filtered by length	
	Forward	Reverse	in combined pool (%)	# of sequences	% recovered	# of sequences	% recovered	# of sequences	% recovered
**S1**	001	008	5	754	12	3,750	99	3,604	96
**S2**	004	010	5	836	13	4,145	99	4,060	98
**S3**	009	014	6	3,558	48	17,532	99	17,202	98
**S4**	012	014	14	7,544	43	37,438	99	36,732	98
**S5**	012	016	5	796	13	3,929	99	3,817	97
**S6**	009	010	16	15,157	76	75,146	99	73,604	98
**S7**	015	016	16	16,169	81	80,165	99	78,265	98
**S8**	004	008	16	15,308	77	75,973	99	74,994	99
**S9**	001	002	17	16,033	76	79,490	99	78,436	99
		**Total:**	**100**	**76,155**	**61**	**377,568**	**99**	**370,714**	**98**

The samples were pooled at ratios that corresponded to the desired sequencing depth. The number and percentage of recovered sequences after each processing step are shown for each sample (S1-S9) and the combined pool (Total).

### Sequencing and raw data processing

Sequencing was carried out on a PacBio Sequel system, using one PacBio flowcell and a collection time of ten hours. Circular consensus sequencing (CCS) was performed on the pooled samples using the SMRT Link software v8.0, producing a consensus sequence for each well of the flowcell. We filtered for sequences that had at least 3 passes and a sequencing accuracy of at least 99% (Q20), which yielded a total of 124,715 sequences.

### Data processing and data analysis

**Figure 3:**
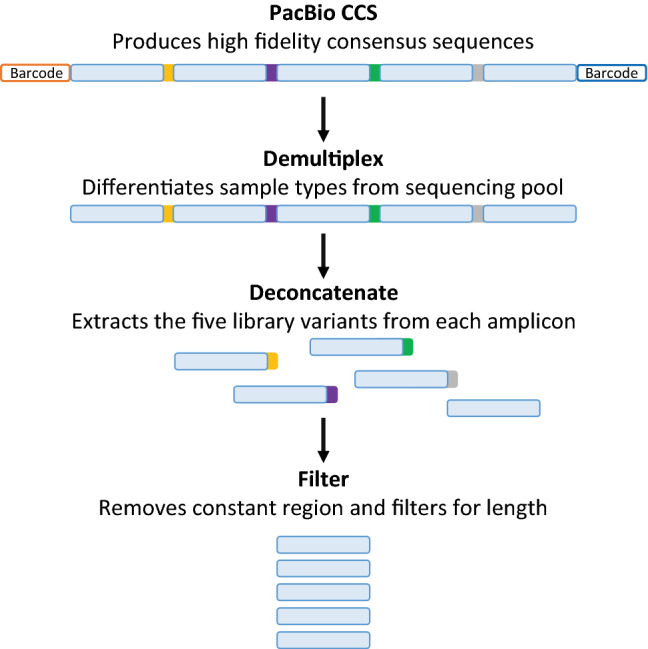
Overview of the post-sequencing data processing. The CCS function in the SMRT Link software v8.0 assimilated the consensus sequences from each well and removed the SMRTbell hairpin adapters. We developed a custom script to sort (demultiplex) and deconcatenate the sequences. Firstly, the sample-specific barcodes were used to separate each selection output from the mixed pool of sequences. Next, we extracted the five original library variants from the concatenated amplicon using the forward and reverse constant region at the 5’ and 3’ termini of each variant (not shown in the figure). A length filter was then applied to ensure the recovery of full-length library variants. See Supplementary Fig. S4 for further detail.

A number of existing bioinformatic tools can perform generic processing of high-throughput DNA sequencing data^51–53^, however, no single tool can demultiplex, deconcatenate and translate DNA sequences to amino acid sequence. Therefore, to process the sequencing data from CCS, we developed DeCatCounter, an all-in-one bioinformatic pipeline for demultiplexing, deconcatenating, filtering by length and (optionally) translating DNA sequencing data (Fig. 3). DeCatCounter takes as input raw, CCS sequencing read files (FASTA or FASTQ) and outputs a dereplicated list of unique nucleic acid sequences and their read counts, together with the associated list of peptides sequences and their read counts, if desired (Fig. 3).

During demultiplexing, DeCatCounter searches each sequence for the sample-specific pair of barcodes and assigns it to the corresponding sample population (see Supplementary Table S2). We allowed for a potential sequencing error of up to two mismatches for each barcode sequence (16 bp) yielding a recovery of 61% of the sequences. Increasing the number of allowed errors to three or four resulted in a similar recovery of 62%. However, decreasing the number of allowed errors to only one mismatch greatly reduced the number of sequences recovered to 50%. We therefore chose to use two allowed mismatches. The retrieval of only 61% of the sequences after the demultiplexing was lower than anticipated. This percentage varied between different samples (Table 1). Demultiplexing retrieved 76–81% of sequences for DNA samples S6-S9; but only 43–48% for S3 and S4 and 12–13% for S1, S2 and S5. The reduced recovery of some samples may be attributable to differences in handling and storage of the fully assembled 5 × amplicons. Samples that were stored for longer time periods and underwent several freeze/thaw events showed the lowest recovery in the demultiplexing step. These factors likely led to damaged flanking regions of the assembled amplicons, which was indicated in the QC assessment of the sequencing run.

To deconcatenate the fully assembled 5 × amplicons into the individual five genes (library variants 1 to 5), DeCatCounter searched for and trimmed the constant regions of 40 bp and 22 bp that flanked each gene on its 5′ and 3′ terminus, respectively (see Supplementary Fig. S4 for details). Certain constant terminal regions are common to gene libraries. Here, these constant termini were part of the original gene library design and had been used to PCR-amplify library variants after each round of selection and directed evolution. For this search, we allowed four potential sequencing mismatches for the 40 bp long 5’-terminal constant region and two mismatches for the 22 bp long 3’-terminal constant region, which also accounts for possible DNA synthesis errors incurred from primer template mismatches and PCR amplification following successive rounds of selection. Applying these constraints resulted in an average of 99% recovery. Increasing or decreasing the number of allowed mismatches by one did not substantially change the recovery rate (1–2% change). Ideally, the deconcatenation step should yield five times as many individual genes compared to the fully sequenced concatemers. Our experimental results demonstrated that this step was highly efficient, yielding 99% of the expected number of genes for all nine sample pools. To ensure that each retrieved sequence represented a gene from the sample populations and not some spurious sequences in the assembly mix, the sequences were filtered for the expected length range between 707–825 bp. This range represents a 5% length variation over the original protein library after the removal of the constant regions used in the demultiplexing process. Variation in length was allowed due to the multiple rounds of directed evolution and homologous recombination experienced by each sample. This filtering for length retained 96–99% of sequences, confirming that our purifications throughout the sample preparation prevented the assembly of unintended sequences (e.g. primers or products of PCR mispriming). The number and percentage of sequences retrieved after each data processing step are detailed in Table 1.

### Analysis of protein populations from directed evolution experiment facilitated by high sequencing depth and accuracy

The nine DNA samples S1-S9 that we sequenced in a single PacBio flowcell were populations of protein variants from a directed evolution experiment. This laboratory selection campaign started from a library based on a single protein containing several randomised regions. Each DNA sample encoded protein variants from different stages or generations of this campaign. The goal was to identify changes in the sequence populations throughout the generations of evolution in response to specific selection pressures.

**Figure 4:**
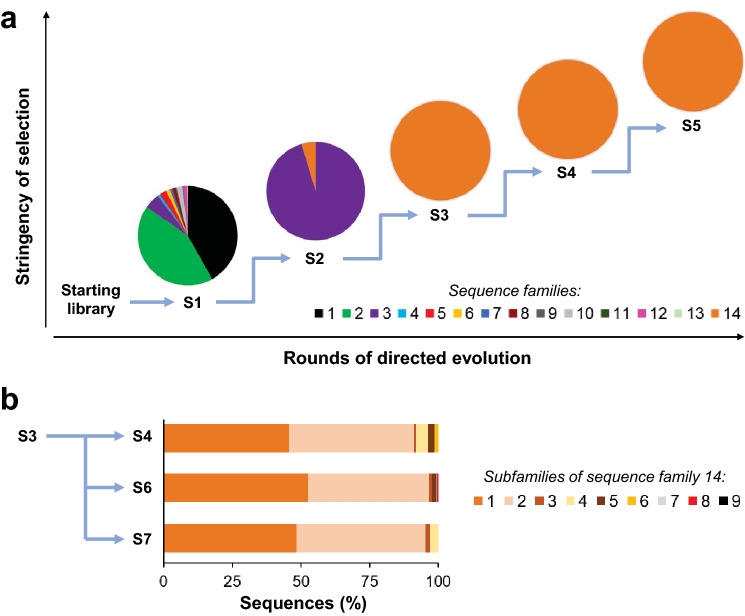
Analysis of nine sequence populations from a protein directed evolution campaign. (**a**) The samples S1-S5 represent protein populations from progressive generations of laboratory evolution performed with stepwise increases in selection pressure. The pie charts for each sample illustrate the distribution of different sequence families within that population. Each family is shown in a different colour and the size of the segment represents the abundance of that family in the sample population. The black, green and purple segments in samples S1 and S2 represent sequence families 1, 2 and 3, respectively. Sequence family 14 is depicted in orange and dominates the sample S3 and all following generations. (**b**) Analysis of sequence distribution at subfamily level for samples S4, S6, and S7. One round of selection was carried out on the same population of protein variants, yet using three different selection conditions resulting in the three sample populations S4, S6 and S7. While all sequences in those samples belonged to sequence family 14, subfamilies were discerned within each of the populations as shown by different shades of orange. These subfamilies constitute either a specific point mutation or a cluster of mutations. The abundance of different subfamilies in a population is represented in the graph. Samples S8 and S9 were experimental control populations and are not shown here.

For each sample population, the demultiplexed, deconcatenated and length-filtered sequences were clustered based on sequence similarity. The clusters (sequence families) were defined by distinct amino acid motifs in the regions of the starting library that had been randomised prior to the directed evolution campaign. Sequence families were translated to their amino acid sequences and ranked based on their relative frequency in their sample population. We then compared the frequencies of protein sequence families within each sample population and across multiple samples. The stringency of the selection conditions had been increased stepwise four times, such that the enriched populations from each selection condition are represented by five samples S1-S5 (Fig. 4a). The population of sequences that resulted from the lowest selection pressure (sample S1) remained highly diverse, with a large number of different sequence families identified. As expected, this diversity was reduced with an increase of selection stringency, as demonstrated by the reduction in the number of sequence families in sample S2. Upon further increase in selection pressure, only a single sequence family appeared to dominate the selection for populations S3-S5. In addition, comparison among samples S1-S3 illustrates how the dominant sequence family in the population changed throughout the course of the evolutionary experiment (Fig. 4a). For example, sequence families 1 and 2 dominated sample S1, accounting for ~ 85% of the population, while family 3 made up only 5% of the population. In contrast, sequence family 3 dominated the population of sample S2, at 95% of the population, while the fraction of sequences belonging to sequence families 1 and 2 were greatly reduced. Starting with sample S2, a new sequence family 14 started growing in frequency. This sequence family 14 became the dominant family for the samples S3-S5.

Finer differences were expected among samples S4, S6, and S7. In contrast to the evolutionary progression of samples S1-S5, these three samples comprised a separate comparative study carried out as three parallel selection branches, all starting from aliquots of sample S3 (Fig. 4b). Each branch was a single round of selection under different selection conditions, yielding samples S4, S6 and S7. Samples S8 and S9 (not shown) were generated as reference samples to control for potential unintended technical bias. As expected, we found the populations of S4, S6 and S7 to also be dominated by sequence family 14, as with sample S3 (Fig. 4). Changes across the three sample populations were therefore more subtle, often differing by only a handful of specific point mutations (see subfamilies in Fig. 4b). These results demonstrated that the sequencing method could resolve unique sequence subfamilies that emerged in the sample population as different selection conditions were applied for just one round of selection. Slight differences between subfamilies could be discerned due to accurate sequencing of each population in sufficient depth, despite using only a single PacBio flowcell for all nine samples.

## Discussion

We developed a method to sequence mid-sized gene populations of > 800 bp to a greater depth and with higher accuracy than has been previously possible without assembly. This method addresses the need for simple and cost-efficient sequencing of mid-sized genes—a need that neither the high-throughput yet short read length Illumina technology, nor the low-throughput yet long-read sequencing technologies can easily address. To analyse and compare several populations of sequences with a length of ~ 870 bp, we created a simple, robust method to increase useful sequence data by concatenating sequences within each population precisely five times. We sequenced the produced concatenated amplicon with the PacBio system. To improve sequencing accuracy up to 99%, we implemented the PacBio circular consensus sequencing (CCS) methodology. This combination of approaches allowed us to fully utilise the maximum sequencing length of the PacBio CCS long-read technology without compromising sequence quality. Previous examples of concatenation resulted in the sequencing of mixed populations due to their inability to control the concatenation or the directionality of genes^39^. Our method improved on these ideas by defining the concatenation extent (fivefold) and enforcing directionality, thereby substantially increasing sequencing data yield and reducing sequencing costs. Furthermore, we designed DeCatCounter, a user-friendly, fast, one-step tool to implement the post sequencing processing of our concatenation method. DeCatCounter takes as input a CCS sequencing read file and outputs a list of unique nucleic acid sequences (and optionally peptide sequences) and their corresponding read counts. DeCatCounter is a Python-based tool and can be run using a single command, which includes values of all user-defined parameters. These parameters include the level of mismatch tolerance used for forward and reverse barcode search, the error tolerance used for forward and reverse constant region search, and the minimum and maximum lengths allowed for the final DNA variants. The barcode sequences used for demultiplexing and the sequences corresponding to the terminal constant regions of a given library used for deconcatenating are provided by the user as additional input text files. To monitor progress, a log file with read counts for each sample along each step of the process is printed as output. In summary, the major practical advantage of our method is the utilization of readily available synthetic biology technology combined with simple, straightforward data processing.

Our concatenation method substantially improved upon the previously published ConcatSeq method^39^ in several aspects. ConcatSeq was designed to sequence shorter sequences of ~ 80–800 bp by concatenating 3 to 50 fragments into amplicons of 300–10,000 bp through Gibson assembly. In the Gibson assembly, the number and direction of concatenations could not be controlled, and a 20 bp region had to be introduced between each concatenated gene. Instead of Gibson assembly, we used the Golden Gate assembly method for a more efficient and controlled concatenation process. The Golden Gate assembly needed only a 4 bp scar sequence to separate each gene. We designed four scars A-D to provide directionality for the assembly of the five library variant pools into amplicons, thereby simplifying and streamlining the subsequent data processing steps. Most importantly, controlling the concatenation improved the sequencing accuracy. The PacBio CCS methodology relies on the ability of the DNA polymerase to sequence an amplicon multiple times using PacBio hairpin adapters^35^. Accurate control of the concatenation enabled us to select the optimal length of the final amplicon (< 5,000 bp) to achieve a desired sequencing accuracy (here, 99% per base). This method improved upon the overall size of amplicons sequenced, their sequencing accuracy and sequencing depth. In contrast to ConcatSeq, the DeCatCounter script demultiplexes the input file according to a provided list of sample-specific barcodes. Our tool allows customization of every parameter and yields a range of useful sequence outputs, including the optional translation to amino acid sequences.

While deconcatenation was 99% efficient, the recovery of sequences during demultiplexing could be improved by reducing degradation of the DNA samples and using maximally distinct barcodes for all samples. The demultiplexing step recovered only 61% of the sequences (Table 1). We noticed that the recovery was highly variable for the different samples S1-S9 and ranged from 12 to 80%. We analysed 28 randomly chosen sequences that failed the demultiplexing step and observed that 18% of these sequences contained barcodes with more errors than the allowed tolerance, 18% were truncated sequences with only a single barcode, and the remaining 64% were short sequence artefacts with repetitive regions. Considering the majority of short sequence artefacts, the quality control (QC) analysis of the PacBio run implied an unusually high level of short sequences, indicating a number of early terminating reads. This finding suggests that the recovery varied across the samples due to different levels of DNA degradation, likely caused by suboptimal handling and storage conditions of individual samples. Samples that had undergone more freeze thaw cycles showed the poorest recovery in the demultiplexing step. Therefore, avoiding such conditions should substantially increase recovery of sequences during demultiplexing. Considering the sequences with problematic barcodes, recovery could be improved further by using unique forward and reverse barcodes for all samples. Each of the nine samples in this study was encoded by two different barcodes, one on each end. However, we used only ten unique barcodes to generate the nine different barcode pairs. For this reason, we could not assign the 18% of sequences that had only one identifiable barcode to a specific sample during the demultiplexing. These sequences could have potentially been recovered if all forward and reverse barcodes had been unique and maximally distinct (18 unique barcodes to encode nine samples). This change alone would have increased the recovery rate from 61 to 77%. The two suggested improvements of avoiding DNA damage and using 18 unique barcodes, taken together, could increase the recovery rate during demultiplexing to > 80%.

We developed this method to enable the sequencing of a mutational library of ~ 870 bp gene variants. We demonstrated that our sequencing method can identify sequence families that represented only a small portion of the total sample population. For example, in sample S1, we identified 13 sequence families, some of them representing as low as 0.3–1% of the sample population (Fig. 4a). In sample S1, sequence family 3 (purple) represented only 5.5% of the population, whereas the same family 3 dominated the population of sample S2 with 95%. Furthermore, family 14 appeared for the first time in sample S2, representing < 5% of the population, but then dominated the selection in all subsequent rounds (samples S3-7). Critically, subtle and quantitative differences could be identified by comparing the sequence traits of sample populations S4, S6, and S7. Each of the sample populations contained subfamilies with small differences consisting of only a few point mutations. Comparisons showed that each of these samples were dominated by two subfamilies that constituted ~ 90% of the population for each sample. However, in the remaining 10% of the population, we observed not only subtle differences in subfamilies across each sample but also the emergence and disappearance of subfamilies. This was possible because the concatenation method enabled a higher sequencing depth than can be achieved by ConcatSeq. The increased depth enabled us to detect minute changes in gene populations after only one round of directed evolution. Downstream data analysis allowed us to observe the convergence of diverse sequence families across evolution, as well as compare the effect of different selection conditions upon a single sequence family.

Our efficient concatenation method is forward-compatible with ongoing hardware improvements to sequencing technology. The recent release of the newest PacBio Sequel II system increased the sequencing capacity of their flow cell by eight-fold. Furthermore, PacBio introduced the Sequel (V3) chemistry that enables High-Fidelity Long Reads, which increased the lifetime of the DNA polymerase to enable sequencing of 10,000–15,000 bp with a sequencing accuracy of 99.9%. Using these hardware improvements in the case of our ~ 870 bp libraries, we would concatenate ten instead of five genes per amplicon, leading to an additional two-fold increase in throughput. These recent advancements in the PacBio technology in combination with our methodology could increase sequencing depth by a total of 16-fold for our medium-length protein libraries. The new High-Fidelity Long-Read chemistry also provides the opportunity to concatenate and analyse protein libraries that contain even longer DNA sequences.

## Conclusions

This method, we believe, describes the most efficient concatenation process performed thus far, producing more sequences of improved quality. We provide a simple and accessible pipeline for utilizing long-read sequencing to compensate for the current limitations of short-read sequencing technology. The appeal of this method lies in the combination of highly optimised technology from synthetic biology with long-read sequencing providing an easy-to-perform platform to sequence medium-length sequences efficiently in a cost-effective manner.

## Materials and methods

All primers were purchased from Integrated DNA Technologies (IDT). Primers containing barcodes (Supplementary Table S2) were purchased with an additional HPLC purification. DNA gel extraction kits were purchased from Qiagen (QIAquick Gel Extraction Kit, 28704), Zymo Research (DNA Clean and Concentrator-5, D4013), and AMPure XP magnetic purification beads (A36881) from Beckman Coulter. Reagents for PCR including dNTPs and Phusion High-fidelity polymerase (E261) were purchased from New England Biolabs (NEB). For the Golden Gate Assembly, the following reagents were purchased: Eco31I (ER0292) and T4 DNA ligase (M1801) from Promega; T7 DNA ligase (L602L) from Enzymatics; BsaI (R0535), BsaI-HFv2 (R3733) and Gate Assembly Kit (E1601) from NEB.

### PCR amplification of sample populations

Each sample population was purified by gel extraction using a 0.8% agarose gel stained with ethidium bromide. The gel bands were excised and purified using a Qiagen gel extraction kit.

Five PCR reactions were carried out for each sample population. Each of the five PCR reactions (1–5) used the complement primers detailed in Supplementary Tables S1 and S2. Primer sets for the amplification of library variants 2, 3 and 4 remained the same for each sample. However, the respective 5′ and 3′ primers containing the barcodes for the amplification of variants 1 and 5 differed for each of the nine sample populations. The names of the barcode-containing primers used to amplify library variants 1 and 5 for each sample are detailed in Table 1 and their sequences are in Supplementary Table S2. A 40 µl PCR reaction was carried out using 175 ng of sample DNA with an initial denaturation of 95 s for 2 min and seven cycles of (95 °C for 60 s, 54 °C for 60 s and 72 °C at 120 s), followed by final extension at 72 °C for 10 min. Residual PCR reagents and PCR primers were removed by AMPure XP beads. AMPure XP beads (50 µl) were added to each PCR reaction and the solution was mixed and incubated at room temperature for 5 min. Supernatant was cleared by placing onto a magnetic stand for at least 3 min. Beads were washed three times with 200 µl of freshly prepared 80% ethanol, and the DNA was eluted using 50 µl of water. The plate was placed onto the magnetic stand until the eluate was separated from the beads. A multichannel pipette was used to carefully extract the eluate from the beads.

### Concatenation of library variants 1 to 5 and PacBio sequencing

Library variants 1 to 5 for each sample were assembled using a one-pot assembly reaction of 100 µl. For each reaction, library variants 1 to 5 (100 ng each) were incubated with 1 µl NEB Golden Gate Assembly Kit BsaI-HFv2 and T4 DNA Ligase buffer as per the manufacturer's protocol. Reactions were kept on ice and transferred to a thermal cycler that was preheated to 37 °C. Assembly reactions were performed by incubating reactions for 5 min at 37 °C and 5 min at 16 °C for 50 cycles, followed by a final incubation step at 55 °C for 5 min (protocol 1; suggested by manufacturer NEB).

Optimization experiments were carried out to assess the assembly efficiency of different reagents. For these experiments, the manufacturer's optimal conditions were followed, and the reactions were performed using three assembly protocols 1–3. Assembly protocol 1 was described above. For assembly protocol 2, reactions were incubated for 1 min at 37 °C and 1 min at 16 °C for 30 cycles, followed by 42 °C for 25 min and 85 °C for 10 min. For assembly protocol 3, we incubated the reactions with 30 cycles of 37 °C for 2 min and 16 °C for 3 min followed by 5 min at 50 °C and 5 min at 80 °C (T. A. Whitehead, personal communication).

Each 20 µl assembly reaction was loaded into a separate well on a 0.6% agarose gel. The gels were run at 80 V for 1 h, allowing for sufficient separation between the fully and partially assembled library variants. Bands were quantified using the ImageJ software. The percentage of the fully assembled 5 × amplicon was calculated from the intensity of the 5 × band relative to the sum of intensities of all bands 1 × through 5 × in that lane. The band representing the fully assembled library variant (5x) was excised and purified using the Zymo DNA clean and concentrator kit, samples were eluted with 20 µl of purified water. Each sample was analysed by NanoDrop photometer to confirm sufficient purity, as indicated by a UV absorption ratio at 260/230 nm of 2.0 or higher. If the ratio was below 2.0, a secondary purification was carried out using AMPure XP beads eluting with no less than 15 µl of purified water.

Each of the assembled amplicons from each sample was analysed by the University of Minnesota Genomics Center by PicoGreen DNA quantification and the Agilent Bioanalyzer for size analysis. The amplicons were then pooled together using the ratios described in Table 1. The volume of the pooled samples was reduced to 40 µl using a pre-cooled SpeedVac concentrator, which is the recommended sample input volume for PacBio sequencing by the UC Davis Genome Center. The final sample was submitted for quality control with PicoGreen quantification and the Agilent Bioanalyzer Supplementary Fig. S3 to ensure no damage upon pooling. The sample (1 µg in 40 µl) was submitted to UC Davis Genome Center.

UC Davis Genome Center ligated the SMRTbell adapters to the 5 × amplicons using PacBio Sequel chemistry (V2). Sequencing was carried out using one flowcell in a PacBio Sequel instrument with a collection time of 10 h. Using the subreads.bam input file, sequences were clustered and quality filtered using the CCS tool from the SMRT Link software v8.0. (Pacific Biosciences). The minimum number of passes was set to three with a minimum predicted accuracy of 99%, which represents a predicted accuracy based on the expected percentage of matches in an alignment of the consensus sequence to the true read. Therefore, only reads expected to be 99% accurate were emitted.

### Data processing

#### Code availability

The Python script DeCatCounter.py was developed to demultiplex, deconcatenate, and filter CCS sequencing data. Source code is freely available on GitHub at https://github.com/ichen-lab-ucsb/DeCatCounter and Dryad at 10.5068/D1V966

#### Data availability

The datasets generated and analysed during the current study are available in their entirety from the corresponding authors on reasonable request. A subsample of the raw PacBio sequencing read files, the barcode sequences and the terminal constant region sequences used for analysis are available on GitHub at https://github.com/ichen-lab-ucsb/DeCatCounter and Dryad at 10.5068/D1V966. Text files with amino acid sequences read counts for the nine DNA library samples resulting from this analysis are also available on Dryad. Sequences in these count files are labelled using the code “sequence_X”, where X corresponds to the order in which the sequence appears in the files. A table of sequence designations and the corresponding amino acid sequence will be given in a future publication.

#### Demultiplexing

Sequencing data were demultiplexed into nine sample populations (S1 to S9) using unique pairs of sample-specific barcodes (Table 1 and Supplementary Table S2). The script searched each sequence for any of the five 16 bp 5’-end forward barcodes (shown in bold and orange in Supplementary Table S2), allowing a tolerance of two errors (mutations, deletions or insertions). If any of these barcodes were identified, the script then searched the sequence for any of the corresponding five 16 bp 3’-end reverse barcodes (shown in bold and blue in Supplementary Table S2), allowing a mismatch of two nucleotides. Sequences for which both a forward and a reverse barcode were identified were classified as belonging to one of the nine sample populations (S1 to S9) and constitute Set 1 (sequence reads in the forward direction, see Supplementary Fig. S4). Similarly, sequences were first searched for the reverse complement of the reverse barcodes at the 5’-end and then searched for the reverse complement of the forward barcodes at the 3’-end. Resulting sequences were classified as belonging to one of the nine sample populations (S1 to S9) and constitute Set 2 (sequence reads in the reverse direction, see Supplementary Fig. S4).

#### Deconcatenation

Amplicons in each sample were deconcatenated into the five individual genes or library variants. For sequencing reads in the forward direction (Set 1), our script searched each sequence for the five 40 bp forward constant regions (5’-CGACTCACTATAGGGACAATTACTATTTACAATTACAATG), allowing an error of four nucleotides. Similarly, for sequencing reads in the reverse direction (Set 2), the script searched for the reverse complement of the five 22 bp reverse constant regions (5’-GGCCAGATCCAGACATTCCCAT), using a tolerance of two mismatches. In both cases, the constant regions were removed from the sequence, resulting in five library variants. Deconcatenated sequences contained approximately 2% artefact sequences that consisted of 1–10 bp at the beginning or the end of the reads, remnants of the SMRTbell adapters attached via A-tailed ligation during library preparation^39^. These short artefact sequences were removed from the data.

#### Trimming constant regions and filtering based on length

For each sequence in Set 1, the script searched and trimmed off the 3’-end reverse constant region (5’-ATGGGAATGTCTGGATCTGGCC), allowing a mismatch of two nucleotides. Similarly, for each sequence in Set 2, the script searched and trimmed off the reverse complement of the forward constant region (5’-CATTGTAATTGTAAATAGTAATTGTCCCTATAGTGAGTCG), allowing four errors. The resulting trimmed variants constitute the individual library variants. To ensure proper full-length sequence in the final processed dataset before downstream analysis, we performed an additional step to retain only sequences of the appropriate length of 707–825 bp.

#### Dereplication to combine set 1 and set 2

For each sample, sequences in Set 2 were converted to the reverse complement and merged with sequences in Set 1. The redundant list of sequences was dereplicated and converted into a list of unique sequences and their associated read counts.

### Data analysis

#### Clustering

Library variants in each sample were clustered into families based on sequence similarity. Sequence similarity was determined by Levenshtein distance (number of substitutions, insertions, or deletions). Every sequence in a family was less than a certain cutoff distance (d_cutoff_) from the centre sequence, which was defined as the most abundant sequence in the family. Samples S1 and S4-S9 were clustered using a distance cutoffs d_cutoff_ = 9, and samples S2-S3 using d_cutoff_ = 5. These specific cutoff distances were empirically chosen based on analysing the number of families as a function of cutoff distances and, ultimately, through visual inspection of the diversity of distinct amino acid motifs in the randomised regions of the starting protein library.

#### Translation and dereplication

Individual sequencing errors at the nucleotide level may lead to inadvertent mistranslation of a DNA sequence. To ensure that closely related sequences (e.g., having sequencing errors) were assigned to the same family at the amino acid level, we first clustered the library variants into families at the nucleotide level and then translated them to amino acids. Dereplication was performed within each family to convert the redundant list of amino acid sequences into a list of unique sequences and their associated read counts. To ensure proper full-length sequences in the final processed dataset, we performed an additional step to retain only sequences of the appropriate length of > 247 amino acids.

## Supplementary Information

Supplementary Information.
