# Supplementary material for: PacBio sequencing output increased through uniform and directional fivefold concatenation
Source: Sci Rep. 2021 Sep 10;11:18065. doi: 10.1038/s41598-021-96829-z (PMC8433307; doi:10.1038/s41598-021-96829-z)
Supplement: Supplementary file 1 — Supplementary Information. [file 41598_2021_96829_MOESM1_ESM.pdf]

Supplementary Information for

PacBio sequencing output increased through uniform and directional 5-fold concatenation

Nisha Kanwar[1,2#*] Celia Blanco[3#], Irene A. Chen[3], & Burckhard Seelig[1,2*]

[1]Department of Biochemistry, Molecular Biology and Biophysics, University of Minnesota, Minneapolis, Minnesota, 55455, USA. [2]BioTechnology Institute, University of Minnesota, St. Paul, Minnesota, 55108, USA. [3]Department of Chemical and Biomolecular Engineering, University of California, Los Angeles, CA 90095

# Co-first authors, * Correspondence to nisha.kanwaruk@gmail.com or seelig@umn.edu.

Variant	Scar		Oligonucleotide sequence (5'-3')
1	n/a	Fwd	See Barcodes
	A	Rev	GCGGCG**GGTCTC**TGCTG*GGCCAGATCCAGACATTCC*
2	A	Fwd	GCGGCG**GGTCTC**ACAGC*CGACTCACTATAGGGACA*
	B	Rev	GCGGCG**GGTCTC**GACAT*GGCCAGATCCAGACATTCC*
3	B	Fwd	GCGGCG**GGTCTC**CATGT*CGACTCACTATAGGGACA*
	C	Rev	GCGGCG**GGTCTC**GCACA*GGCCAGATCCAGACATTCC*
4	C	Fwd	GCGGCG**GGTCTC**CTGTG*CGACTCACTATAGGGACA*
	D	Rev	GCGGCG**GGTCTC**ATTGC*GGCCAGATCCAGACATTCC*
5	D	Fwd	GCGGCG**GGTCTC**TGCAA*CGACTCACTATAGGGACA*
	n/a	Rev	See Barcodes

Supplementary Table S1. Oligonucleotides used for the five PCR reactions per sample to prepare the library variants for the concatenation procedure. Bsa I restriction sites are in bold and underlined, the unique 4 bp scar regions are highlighted in colour, and gene-overlapping regions are shown in italics.

Variant	Barcode Number		Oligonucleotides used to attach barcodes (5'-3')
1	001	Fwd	**CACATATCAGAGTGCG***CGACTCACTATAGGGACA*
	004		**CACGCACACACGCGCG***CGACTCACTATAGGGACA*
	009		**ACACACGCGAGACAGA***CGACTCACTATAGGGACA*
	012		**ACACTAGATCGCGTGT***CGACTCACTATAGGGACA*
	015		**CGCATGACACGTGTGT***CGACTCACTATAGGGACA*
5	002	Rev	**ACACACAGACTGTGAG***GGCCAGATCCAGACATTCC*
	008		**ACAGTCGAGCGCTGCG***GGCCAGATCCAGACATTCC*
	010		**ACGCGCTATCTCAGAG***GGCCAGATCCAGACATTCC*
	014		**CTCACTACGCGCGCGT***GGCCAGATCCAGACATTCC*
	016		**CATAGAGAGATAGTAT***GGCCAGATCCAGACATTCC*

Supplementary Table S2. Oligonucleotides used to attach the sample-specific barcodes. The barcode-encoding regions are shown in bold and colour. The regions overlapping with the termini of the concatenated amplicon are shown in italics. The oligonucleotides attach the barcodes either to the 5'-end of library variant 1 (Fwd primer, orange) or to the 3'-end of library variant 5 (Rev primer, blue).

Sample	S4		S7		S2
**Total DNA after PCR (µg)**	0.62		1.04		2.24
**Original template (%)**	28		17		8

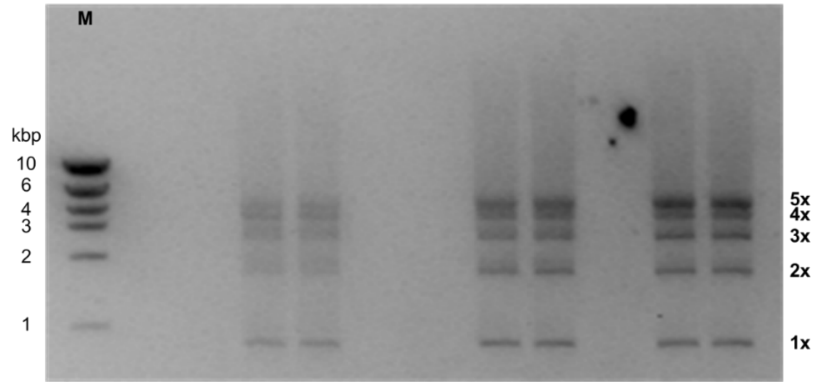

Supplementary Figure S1. Initial attempts at the concatenation showed different efficiencies of assembly for three different samples. Improved efficiency correlated with increased PCR yield of the samples. The total DNA after PCR pertains to the DNA recovered after PCR amplification of the 5x amplicons 1-5 for each sample calculated as an average of the two identical repeats. As PCR yield increased, the assembly efficiency increased because the percentage of original template DNA was reduced. A DNA marker as a size reference was loaded in the leftmost lane (M).

BsaI (NEB)	+				+								
BsaI HF v2 (NEB)						+	+						
Eco31I (Promega)								+		+		+	
T4 DNA Ligase (Promega)	+				+	+	+						
T7 Ligase (Enzymatics)								+		+		+	
Golden Gate kit (NEB)			+	+					+		+		+
Enzymes 3-fold increased								+	+				
Fully assembled amplicon (%)	7		40	25	4	27	18	4	36	1	2	1	31

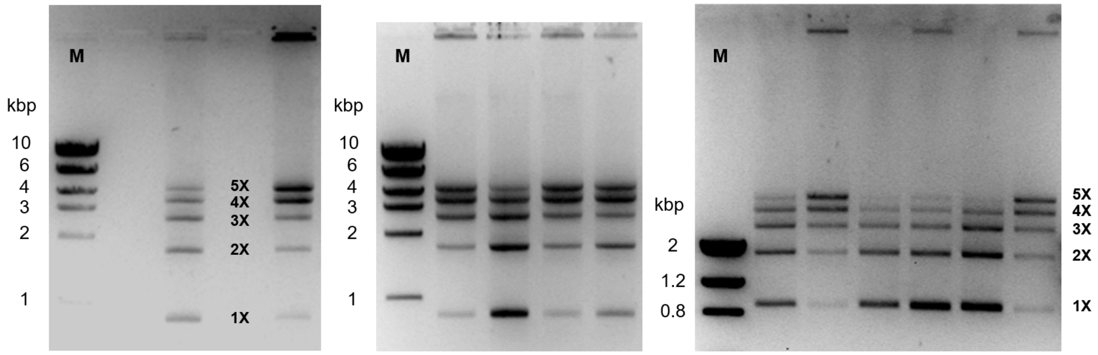

Supplementary Figure S2. Optimizing the efficiency of the assembly process. The efficiency of concatenating the five library variants was compared for assemblies using different commercially available enzymes and following protocols 1-3 (black, green and orange, respectively; see methods section). Optimal performance was determined by the percentage of the fully assembled 5x product (between 1% and 40%). The most efficient assembly was achieved with the NEB Golden Gate assembly kit and protocol 1 (increased reaction time and number of cycles). Further details can be found in the methods section. The lane containing a molecular mass ladder is labelled M in each gel.

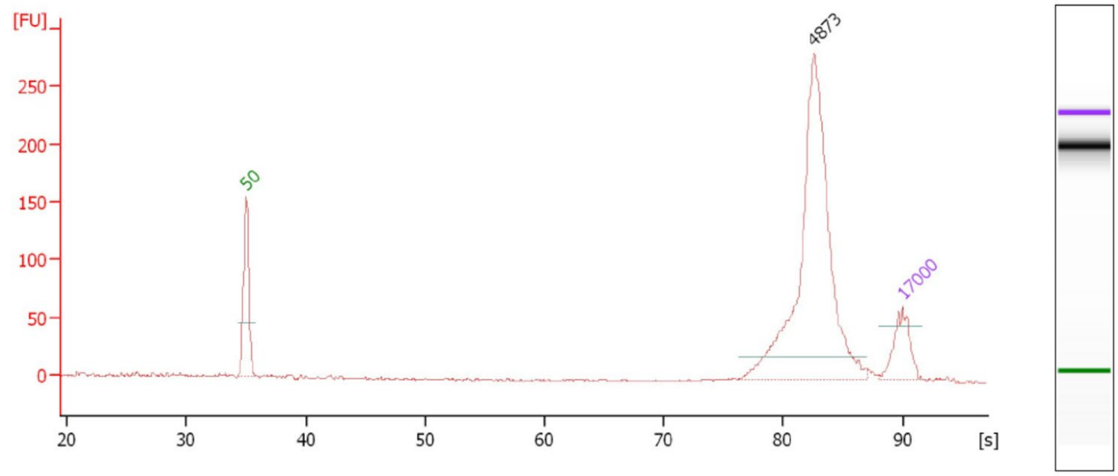

Peak		Size [bp]	Conc. [ng/µl]	Molarity [nmol/l]	Observations
1		50	8.30	251.5	Lower Marker
2		4,873	35.95	11.2	
3		17,000	4.20	0.4	Upper Marker

Supplementary Figure S3. Analysis of size distribution by Agilent Bioanalyzer of the pooled concatenated amplicons S1-S9 directly before submission for sequencing.

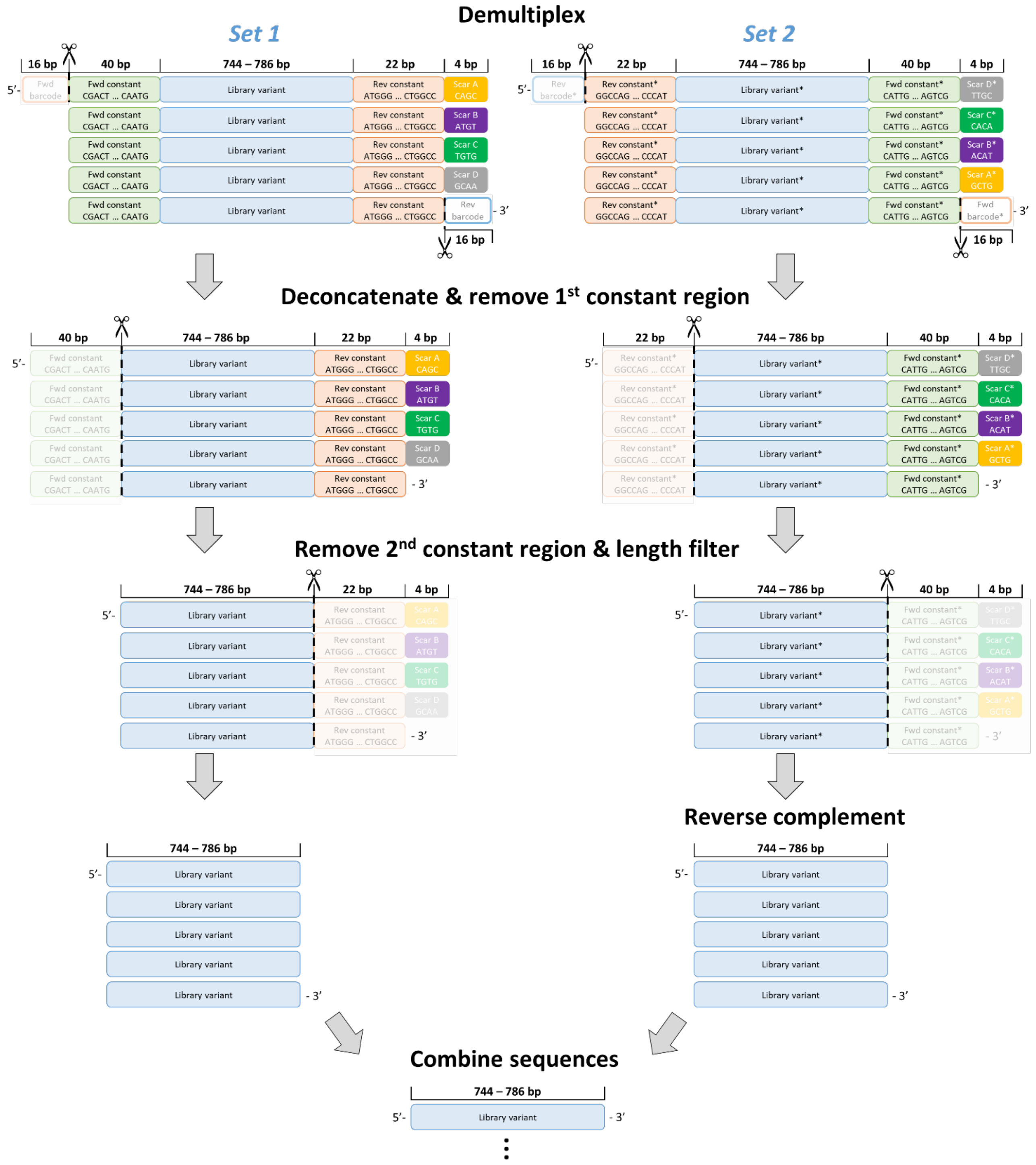

Supplementary Figure S4. Detailed schematic representation of the data processing workflow to extract individual library variants (gene sequences) from the PacBio sequencing run. PacBio can read a sequence from either direction and therefore produces two sets of amplicons: one as the sense strand and the other as the reverse complement (Set 1, Set 2). Our sequencing output represents a pool of different samples that each contain a unique pair of 16 bp sequencing barcodes flanking the fully assembled 5x amplicon. Therefore, amplicons were

first demultiplexed by identifying their pair of barcodes and assigning them into sample specific sub-pools accordingly. Each sub-pool was then deconcatenated to extract the five individual library variants contained within each 5x amplicon. As each sequence can be sequenced in the forward or reverse direction, sequences were scanned for either of the two constant regions that flank each variant and the first identified constant region was removed. Each sequence was trimmed by identifying and removing the opposing flanking region (including the second constant region), and filtered by size using a 5% error margin of the expected size range (707-825 bp). Finally, the library variants from reads in the reverse direction were reverse-complemented and merged with the forward reads. The library variants from each sample were subsequently clustered into families of similar sequences, translated to amino acid sequences, and dereplicated within each family (not shown in figure).
